# Multiple forms of discrimination and postpartum depression among indigenous Palestinian-Arab, Jewish immigrants and non-immigrant Jewish mothers 

**DOI:** 10.1186/s12889-019-8053-x

**Published:** 2019-12-27

**Authors:** Nihaya Daoud, Neveen Ali Saleh-Darawshy, Meiyin Gao, Ruslan Sergienko, Stephanie Ruth Sestito, Nabil Geraisy

**Affiliations:** 1 Department of Public Health, School of Public Health, Faculty of Health Sciences, Ben-Gurion University of the Negev, P.O. Box 653, 84015 Beer Sheva, Israel; 2 Critical Care Services Ontario, LuCliff Place, 700 Bay Street, Suite 1400, Toronto, ON M5G 1Z6 Canada; 3 School of Medicine, Faculty of Health Sciences, Ben-Gurion University of the Negev, P.O.28 Box 653,, 84015 Beer Sheva, Israel; 4 Department of Psychiatry, Nazareth Hospital, E.M.M.S, Nazareth, Israel

**Keywords:** Discrimination, Multiple forms of discrimination, Ethnic discrimination, Postpartum depression, Minority, Immigrant, Palestinian-Arabs, Jewish immigrants, Israel

## Abstract

**Background:**

While discrimination takes multiple forms, racial or ethnic discrimination is a root cause of this health-damaging social phenomenon. We drew on intersectionality theory, which offers an account of discrimination’s multiple effects, to consider associations between women’s experiences of discrimination and postpartum depression (PPD) using four measures: single forms of discrimination (SFD); multiple forms of discrimination (MFD); ethnic discrimination combined with MFD (E-MFD); and a composite MFD that interacted with women’s identity (C-MFD).

**Methods:**

We interviewed a stratified sample of 1128 mothers face to face in 2014–2015 during mothers’ visits to maternal and child health clinics. The mothers belonged to three groups in Israel: Palestinian-Arab minority, Jewish immigrant, and non-immigrant Jewish. We conducted unadjusted and adjusted logistic regressions for PPD, measured on the Edinburgh Postnatal Depression Scale, in associations with SFD (experiencing discrimination based on any of the following: age, sex, class, ethno-national identity, religiosity level and skin color); MFD (experiencing 0,1, 2 or ≥ 3 of SFD); E-MFD (ethnic discrimination combined with other MFD); and finally, C-MFD (interaction between MFD and women’s identity).

**Results:**

Palestinian-Arab mothers had higher PPD and reported higher SFD (based on ethnicity, religiosity level, and socioeconomic status), as well as higher MFD and E-MFD. This was followed by Jewish immigrant mothers, and lastly by non-immigrant Jewish mothers. However, both MFD and E-MFD had a strong association with PPD among non-immigrant Jewish mothers reporting 2MFD and ≥ 3MFD, and Palestinian-Arab mothers reporting ≥3MFD, but no significant association among immigrant Jewish mothers. When we used C-MFD, we found a dose-response association in which Palestinian-Arab mothers experiencing more MFD (2MFD and ≥ 3MFD) were more likely to experience PPD. This was followed by immigrant Jewish mothers (reporting 2MFD and ≥ 3MFD), and lastly by non-immigrant Jewish mothers.

**Conclusions:**

MFD should be considered in relation to women’s identity (being part of a minority, immigrant, or non-immigrant majority group) in maternal mental health research and practice. Otherwise, we risk underestimating the effects of MFD on PPD, especially in minority and immigrant mothers, who are more likely to face interlocking forms of discrimination.

## Background

Discrimination is a malicious feature of societies that is rooted in racist ideology and colonization [1]. Discrimination systemically classifies people into groups, perpetuates uneven distribution of power and privileges, and maintains the superiority of some groups over others [2, 3]. Discrimination leads to differential institutional policies that encourage certain attitudes, behaviors, acts, and unfair treatments [4]. Institutional discrimination can thus authorize interpersonal discrimination based on stereotypes about the characteristics, abilities, motives, and intentions of particular groups [5–7].

While racial or ethnic exclusion is at the core of discrimination (racism), other forms of discrimination can co-occur, including ageism, sexism, classism, etc. [2, 6, 8, 9]. Intersectionality theory was developed in the US by Black feminists precisely in order to address the effects of this co-occurrence on women’s social status and health [10, 11]. According to intersectionality, when ethnic discrimination combines with one or more other form of discrimination (i.e., multiple discrimination). some people are exposed to discrimination in many domains (individual, institutional, cultural) of life simultaneously. These domains intertwine to create a system of oppression [12] and marginalization [10, 11, 13, 14]. Thus, participating in more than one marginalized axis of identity can amplify discrimination, for example, being an ethnic or racial minority and a woman of color; being of older age and living in a low socioeconomic-status area. In this view, multiple forms of discrimination (MFD) can generate “toxic situations” that increase stress and activate a chain of psychophysiological processes that damage health [8, 10, 15–18].

To date, most research on intersectional effects on health has relied on qualitative methods [10, 19, 20], while few studies have used quantitative methods [10]. As well, research sometimes focuses on a single form of discrimination (SFD). But this might fail to capture the complex picture of MFD, leading to underestimation of the full effect of discrimination on health [14, 19]. Studies focused on racial or ethnic discrimination only, leaving out the interrelated effects of other MFD, might underestimate the effects on health of ethnic and racial discrimination as it interlocks with other forms [19]. Similarly, measuring a cumulative (additive) effect of different forms of discrimination cannot capture the effect of the full spectrum of MFD on health if the measure ignores those interlocking effects between ethnic identity and MFD [10, 19]. And while previous research showed that racial or ethnic discrimination was associated with poor mental and physical health [21–23], promising emerging research has identified a dose-response association between multiple discrimination and poor self-rated health and mental health [8, 15, 17], and facing more forms of discrimination was associated with poorer health and depression [15, 24, 25].

### Discrimination and postpartum depression (PPD)

Previous research, mainly from Western countries (the US, Europe or New-Zealand), showed that discrimination is associated with increased risk of health problems during pregnancy and after birth [26], including postpartum depression (PPD) [27–29]. PPD is a serious public health issue faced by almost one fifth of new mothers in Western countries [30] and up to two thirds in non-Western countries [31]. Likely to occur 6 weeks to 6 months after birth, PPD is characterized by a wide range of symptoms, including loss of pleasure in life, despair, crying, and even suicidal thoughts [32, 33]. These can impair mothers’ daily functioning and lead to self-harm and, in some cases, harm to babies [34]. PPD is highly prevalent among Indigenous minority and immigrant mothers [35, 36].

Discrimination might act via different mechanisms that lead to, or intensify mental health problems among new mothers in these groups (e.g., high stress, low social support, low socioeconomic status) [26, 37], as they are likely to face MFD [24]. This discrimination takes the form of denial of basic human and women’s rights [10, 18], fewer opportunities for higher education and income, and increased violence, including intimate partner violence [38, 39]. In addition, these women lack social support [40], are likely to live in areas lacking services and resources, and face increased barriers in accessing health care and social services for PPD treatment [41]. To date, however, few studies have put intersectionality to use in studying associations between MFD and PPD, and no study that we know of has compared Indigenous ethnic minority women, immigrant women, and non-immigrant women in this regard.

### Discrimination and PPD in Israel

In Israel, no research has examined relationships between discrimination and PPD. One recent study found ethno-national inequalities in PPD, showing higher prevalence among Palestinian-Arab women compared to Jewish women (20.8% vs. 7%) [36]. Risk factors associated with PPD in these two ethno-national groups of women might reflect their unequal social, economic, and political positions in Israeli society. While both groups suffer ramifications of the prolonged political Israeli-Palestinian conflict [42, 43], Palestinian-Arabs also live with discriminatory institutional policies [44, 45] and stigma [46]. Palestinian-Arabs are an Indigenous minority in Israel who make up about one fifth of the population [47]. Today, they are in a crisis situation vis-a-vis the state [43]. After years of restricted socioeconomic development [48], educational and work opportunities [44, 49, 50], and amidst growing income inequalities [51], Palestinian-Arabs are socially and politically excluded from power [43, 52]. The position of Palestinian-Arabs in relation to the majority Jewish group, and emerging from their complex ethno-national background (Palestinian-Arab), citizenship status (Israeli), cultural background (traditional, patriarchal), religion (Muslim, Christian, Druz) and level of religiosity (traditional, religious, or secular), might open this group to experiencing MFD.

The other largest group of interest in the current study is immigrant Jewish women. In Israel, descendants of the Jewish population that immigrated to the country after its founding in 1948 are full citizens [42]. So are foreign-born Jews, who make up a quarter of the country’s population [47]. These immigrant Jews arrived in large waves after 1948 from North Africa, Arab countries, North America, and Europe. Their presence has led to a delineation of two Jewish ethnic identities in Israel: Mizrahim, from Arab countries, and Ashkenazim, from Europe and North America [53]. A later wave of immigrants arrived in the 1990s from former Soviet states and Ethiopia, causing further ethno-cultural fragmentation in Jewish Israeli identity. Today, immigrant Jews are less integrated into the society and political leadership. Yet they share the view that Israel’s identity must have a strong Jewish component, effectively leaving out Palestinian-Arab citizens [42]. This has important implications for Palestinian-Arab citizens in terms of multi-level discrimination based on their ethno-national identity and other identities [42, 52]. Nonetheless, Palestinian-Arabs, Mizrahi Jews and Ethiopian immigrant Jews are the most stigmatized groups in Israel society [46].

Previous research on discrimination in Israel might has failed to fully account for discrimination’s effects on health. While experiences of interpersonal and institutional discrimination have been associated with poor mental health and unhealthy behaviours among Palestinian-Arab minority men [22, 54], this association has not been consistent [55]. For example, Palestinian-Arab citizens and immigrant Jews in Israel reported higher discrimination compared to others in one study, but discrimination was associated with poor mental and physical health only among non-immigrant Jews [56]. As for PPD, no studies in Israel have examined its associations with discrimination. Studies on inequalities in PPD prevalence and risk factors among ethno-national groups of mothers [36], religious groups of women in Israel [57], and levels of barriers to PPD care [41] suggest intersections of different forms of discrimination emanating from ethno-national and cultural identity, level of religiosity, socioeconomic status [54], and immigrant status, and that this discrimination transforms into ethno-national and gender inequities among women in different segments of Israeli society.

To examine this more deeply, we compared PPD among women with different experiences of discrimination, which we determined by four measures of discrimination: single forms of discrimination (SFD), multiple forms of discrimination (MFD), ethnic discrimination with multiple forms of discrimination (ethnic and MFD combined, or E-MFD) and composite multiple forms of discrimination (C-MFD), a composite variable that included interactions between women’s identity and MFD. We compared these associations among three groups of women in Israel: Palestinian-Arab, Jewish immigrant, and non-immigrant Jewish. We hypothesized that:

1. Palestinian-Arab women would report higher forms of discrimination (SFD, MFD, E-MFD), while Jewish immigrant women would report second-highest MFD, and non-immigrant Jewish women would report lowest forms of discrimination;
2. MFD and E-MFD would be associated with PPD in a dose-response manner among all groups, and this association would follow the same order as above;
3. The association between the C-MFD and PPD would be stronger than the association between the SFD, MFD, E-MFD and PPD; and
4. The C-MFD would show a stronger association with PPD among Palestinian-Arab women, followed by Jewish immigrant women, and lastly by non-immigrant Jewish women.

## Methods

### Data and study population

We used cross-sectional data from the “Family Relations, Violence and Health.” The current analysis included 1128 mothers who were 6 weeks to 6 months after birth [38, 58]. Data collection for the original study took place from October 2014 to October 2015, and included a stratified sample of eligible Palestinian-Arab and Jewish mothers (≥26 weeks of pregnancy, and 6 weeks to 6 months after birth). In total, 1401 women were interviewed face to face while visiting 63 maternal and child health (MCH) clinics in five regions of Israel. The number of participating women and clinics was determined by the proportion of births in a region and women’s ethnicity (Palestinian-Arab vs. Jewish). Trained female interviewers invited eligible mothers visiting participating MCH clinics to take part in the study. Women who agreed were invited into a separate room where the interviews were conducted after obtaining a signed informed consent form. Interviews were based on a structured questionnaire in Arabic or Hebrew. As part of a national PPD screening program among pregnant and postpartum mothers in Israel, all study participants were screened for PPD at least one time by a MCH nurse before they were asked about PPD symptoms by the study interviewers. Among the total sample, at the time of the interview, 1128 women were 6 weeks to 6 months after birth; these women were included in the current analysis. The response rate for the original sample was 76% for Palestinian-Arab women, 73% for Jewish women.

### Study measures

#### Postpartum depression (PPD)

the main health outcome, was measured using the Edinburgh Postnatal Depression Scale (EPDS), which evaluates a woman’s feelings over the past week and is sensitive for identification of PPD [59]. The Hebrew [60] and Arabic [61] versions of the EPDS have high sensitivity and specificity. Cronbach’s alpha for the EPDS in the current study was 0.82. The EPDS includes 10 questions, with response categories ranging from 0 to 3. The total score ranges from 0 to 30. A score of 10 or more, which we used in the current analysis as a cutoff for PPD, had been considered a risk for PPD in previous research [35, 36, 59].

#### Women’s study groups or identity

was determined by a combination of answers to questions about: women’s ethno-national identity (self-identified measure as Palestinian-Arab or Jewish) and immigrant status (a direct question about the woman’s country of birth: Israel or a foreign country). Answers revealed three groups of women: Palestinian-Arab, Jewish immigrant, and non-immigrant Jewish.

#### Single forms of discrimination (SFD)

was measured using a question on experiencing any of 6 forms of discrimination, as follows: “How often do you feel that you are being discriminated against in Israeli society based on: 1. your ethnicity or nationality; 2. your gender; 3. your skin color; 4. your age; 5. socioeconomic position (SEP) or class; and 6. your level of religiosity.” Answer categories included: 1. Often, 2. Sometimes, 3. Seldom, and 4. Never. We dichotomized answers into ‘yes’ (including the first three categories for experiencing any of these SFD) and ‘no’ (never experiencing any of these SFD).

#### Multiple forms of discrimination (MFD)

Was measured by the sum score of answers to questions on the 6 SFD, with answers categorized according to the number of SFD a woman reported: none, 0, 1, 2, and ≥ 3 forms of discrimination

#### Ethnic and multiple forms of discriminations (E-MFD)

was a combined measure for reporting discrimination based on a woman’s ethnic or national background in addition to other MFD, resulting in the following categories: 1. none, 2. ethnic only, 3. ethnic and 1SFD, 4. ethnic and 2SFD, 5. ethnic and ≥ 3SFD, and 6. other forms of discrimination (not ethnic).

#### Composite-MFD (C-MFD)

was a composite variable resulting from interactions between the study groups (Palestinian-Arab, Jewish immigrant, and non-immigrant Jewish women) and experiencing MFD (0,1,2, ≥3). This yielded 12 categories as follows: Palestinian-Arab ≥3SFD, Palestinian-Arab 2SFD, Palestinian-Arab 1SFD, and Palestinian-Arab reporting no discrimination; Jewish immigrant ≥3SFD, Jewish immigrant 2SFD, Jewish immigrant 1SFD, and Jewish immigrant reporting no discrimination; and non-immigrant Jewish ≥3SFD, non-immigrant Jewish 2SFD, non-immigrant Jewish 1SFD, and non-immigrant Jewish reporting no discrimination as a reference category.

#### Other covariates

were variables studied in previous research on discrimination and PPD [16, 17, 62], including age (16–24, 25–34, and 35–48), marital status (married or not married, including single, divorced, separated or widowed), work status (working or not working), number of children (categorized into 0–1, 2–3, and 4–12), and antidepressant use (yes/no).

#### Statistical analysis

We conducted the analysis using SPSS version 25. After data cleaning we found very few missing data (less than 4%). The first step of the analysis was to compare prevalence of PPD by study group (Palestinian-Arab, Jewish immigrant, and non-immigrant Jewish) and by socio-demographic characteristics. After creating the discrimination measures of SFD, MFD, E-MFD and C-MFD, we examined the associations between these variables and PPD. We studied these univariate associations using Chi-square test. Since we aimed to compare the association of each of the discrimination variables (SFD, MFD, E-MFD) with PPD for each study group, we conducted multivariable logistic regression analysis using generalized estimation equation (GEE) procedure to consider the MCH clinic cluster effect and calculate the odds ratios and 95% confidence intervals for the associations between each of the SFD, MFD, E-MFD and PPD for women in each study group. We fitted two models: the first model was unadjusted, and the second model was adjusted for independent variables that were associated with PPD in the univariate associations (age, education, work status, and antidepressant use).

Lastly, as we were interested in the interlocking associations between MFD and identity, we continued our analysis with multivariable analysis of PPD (using logistic regression and GEE procedure) while considering the composite variable of C-MFD. Here, we also fitted two multivariable models: one unadjusted and one adjusted for independent variables of age, education, work status, and antidepressant use. To explore any possible multicollinearity, we examined correlations between the study independent variables before conducting the multivariable analysis. All coefficients were lower than our threshold of 0.4 (Appendix 1), and none of the variables was excluded in the multivariable analysis. Level of significance was set to 5% in the study.

## Results

Our study sample included 1128 postpartum mothers (276 Palestinian-Arab, 221 Jewish immigrant and 632 non-immigrant Jewish). Palestinian-Arab mothers were younger, more likely to be married, more likely to be unemployed, and had lower education than the two groups of Jewish women. More Palestinian-Arab women reported a traditional level of religiosity and having more children, but they were less likely to use antidepressants compared to the other two groups of mothers (Table 1).

**Table 1:** Distribution of study variables by study group and associations with postpartum depression (PPD)

	Total	Non-Immigrant Jewish	Jewish Immigrant	Palestinian-Arab		Postpartum Depression (PPD)	
Total	N (%)	N (%)	N (%)	N (%)	*p*	N (%)	*p*
	1128 (100)	632 (56.0)	221 (19.6)	275 (24.4)		116 (10.3)	
Study group							
Non-Immigrant Jewish						39 (6.2)	*< 0.001*
Immigrant Jewish						20 (9.1)	
Palestinian-Arab						57 (20.8)	
Age							
16–24	169 (15.0)	54 (8.5)	20 (9.1)	95 (34.7)	*< 0.001*	34 (20.1)	*<.0001*
25–34	701 (62.2)	398 (63.0)	155 (70.1)	148 (54.0)		56 (8.1)	
35–48	257 (22.8)	180 (28.5)	46 (20.8)	31 (11.3)		26 (10.2)	
Marital status							
Married	1063 (94.3)	592 (93.8)	201 (91.0)	270 (98.2)	*0.002*	109 (10.3)	*0.875*
Not married	64 (5.7)	39 (6.2)	20 (9.0)	5 (1.8)		7 (10.9)	
Education							
BA MA & PhD	544 (48.2)	353 (55.9)	117 (52.9)	74 (26.9)	*< 0.001*	38 (7.0)	*< 0.001*
Postsecondary Education	201 (17.8)	111 (17.6)	48 (21.7)	42 (15.3)		14 (7.0)	
High School or Less	383 (34.0)	168 (26.6)	56 (25.3)	159 (57.8)		64 (16.9)	
Employment Status							
Yes	680 (60.7)	466 (74.2)	141 (64.4)	73 (26.6)	*< 0.001*	54 (8.0)	*0.002*
No	441 (39.3)	162 (25.8)	78 (35.6)	201 (73.4)		61 (13.9)	
Religiosity level							
Religious	284 (25.2)	174 (27.5)	39 (17.7)	71 (25.8)	*< 0.001*	31 (11.0)	*0.626*
Traditional	439 (39.0)	218 (34.5)	71 (32.3)	150 (54.6)		48 (11.0)	
Not Religious	404 (35.8)	240 (38.0)	110 (50.0)	54 (19.6)		37 (9.2)	
Number of Children							
0–1	398 (35.3)	219 (34.7)	84 (38.0)	95 (34.6)	*0.015*	44 (11.1)	*0.241*
2–3	571 (50.7)	322 (51.0)	120 (54.3)	129 (46.9)		51 (9.0)	
4–12	158 (14.0)	90 (14.3)	17 (7.7)	51 (18.6)		21 (13.3)	
Antidepressant use					*< 0.001*		
Yes	15 (1.3)	6 (0.9)	7 (3.2)	2 (0.8)		6 (40.0)	*0.001*
No	1101 (98.7)	625 (99.1)	214 (96.8)	262 (99.2)		108 (9.9)	

PPD prevalence was significantly higher (20.8%) among Palestinian-Arab women, followed by Jewish immigrant women (9.1%), then non-immigrant Jewish women (6.2%). The prevalence of PPD was also higher among women in the youngest age group (16–24 years), those with lower education (high school or less), unemployed women, and those who had used antidepressants (Table 1).

Tables 2 shows the distribution of SDF, MFD and E-MFD among the study groups and associations between the measures of discrimination and PPD. Compared to the Jewish mothers’ groups, Palestinian-Arab women reported significantly higher SFD grounded in their ethno-national identity (55.8%), socioeconomic status (17.5%), and religiosity level (35.2%). Jewish immigrant women reported the next-highest SFD, where 45.9% reported ethno-national discrimination, 10.1% socioeconomic discrimination and 21.8% religiosity-level discrimination. Last were non-immigrant Jewish mothers, who reported lower levels of discrimination based on ethno-national identity (18.6%), socioeconomic status (8.9%), and religiosity level (16.9%). However, there were no significant differences between the study groups regarding other forms of discrimination (based on age, gender or skin color), although Palestinian-Arab women reported higher levels of skin-color discrimination.

**Table 2:** Experiences of SFD, MFD and E-MFD by study group and univariate associations between discrimination variables and postpartum depression (PPD)

	Total	Non-Immigrant Jewish	Jewish Immigrant	Palestinian-Arab		Postpartum Depression (PPD)	
Total	N (%)	N (%)	N (%)	N (%)	*p*	N (%)	*p*
	1128 (100)	632 (56.0)	221 (19.6)	275 (24.4)		116 (10.3)	
Single forms of discrimination (SFD)							
Discrimination based on ethno-national identity							
Yes	372 (33.1)	117 (18.6)	101 (45.9)	153 (55.8)	*< 0.001*	61 (16.5)	*< 0.001*
No	752 (66.9)	513 (81.4)	119 (54.1)	121 (44.2)		55 (7.3)	
Discrimination based on socioeconomic status							
Yes	126 (11.3)	56 (8.9)	22 (10.1)	48 (17.5)	*0.001*	30 (23.8)	*< 0.001*
No	994 (88.8)	572 (91.1)	196 (89.9)	226 (82.5)		86 (8.7)	
Discrimination based on religiosity level							
Yes	250 (22.3)	106 (16.9)	48 (21.8)	96 (35.2)	*< 0.001*	41 (16.4)	*0.001*
No	872 (77.7)	523 (83.2)	172 (78.2)	177 (64.8)		75 (8.7)	
Discrimination based on age							
Yes	117 (10.4)	67 (10.6)	21 (9.5)	29 (10.6)	*0.896*	22 (18.8)	*0.002*
No	1007 (89.6)	563 (89.4)	199 (90.5)	245 (89.4)		94 (9.38)	
Discrimination based on gender							
Yes	449 (39.9)	263 (41.7)	89 (40.5)	96 (35.0)	*0.163*	57(12.75)	*0.033*
No	675 (60.1)	367 (58.3)	131 (59.6)	178 (65.0)		59 (8.78)	
Discrimination based on skin color							
Yes	106 (9.4)	48 (7.6)	25 (11.4)	33 (12.1)	*0.060*	21 (19.81)	*< 0.001*
No	1017 (90.6)	582 (92.4)	195 (88.6)	240 (87.9)		94 (9.29)	
Multiple forms of discrimination (MFD)							
None	467 (41.5)	290 (46.0)	79 (35.9)	98 (35.8)	*< 0.001*	28 (6.0)	*< 0.001*
1 form	245 (21.8)	153 (24.3)	47 (21.4)	45 (16.4)		18 (7.4)	
2 forms	167 (14.9)	75 (11.9)	41 (18.6)	51 (18.6)		25 (15.2)	
≥ 3 forms	245 (21.8)	112 (17.8)	53 (24.1)	80 (29.2)		45 (18.4)	
Ethnic discrimination combined with MFD (E-MFD)					*< 0.001*		*< 0.001*
None	467 (41.5)	290 (46.0)	79 (35.9)	98 (35.8)		28 (6.0)	
Ethno-national exclusive	59 (5.2)	11 (1.7)	17 (7.7)	31 (11.3)		6 (10.2)	
Ethno-national and 1 other SFD	111 (9.9)	29 (4.6)	35 (16.9)	47 (17.2)		18 (16.5)	
Ethno-national and 2 other SFD	91 (8.1)	27 (4.3)	27 (12.3)	37 (13.5)		13 (14.3)	
Ethno-national and ≥ 3 SFD	111 (9.9)	50 (7.9)	22 (10.0)	39 (14.2)		24 (21.6)	
Other discrimination (not ethno-national)	285 (25.4)	223 (35.4)	40 (18.2)	22 (8.0)		27 (9.5)	

Regarding MFD, Palestinian-Arab women were more likely to report having experienced ≥3 forms (29.2%), followed by Jewish immigrant women (24.1%), then non-immigrant Jewish women (17.8%). Experiencing 2 forms of discrimination was similar among Palestinian-Arab mothers and Jewish immigrant mothers (18.6%), and lower among non-immigrant Jewish mothers (11.9%). Experiencing 1 form of discrimination was highest among non-immigrant Jewish women (24.3%), followed by Jewish immigrant women (21.4%), and lowest (16.4%) among Palestinian-Arab women. Also, non-immigrant Jewish women were most likely to report no discrimination (46%) compared to the other two groups of Jewish immigrant and Palestinian-Arab women (35.9 and 35.8%, respectively).

With regards to E-MFD, Palestinian-Arab women most often reported exclusively ethno-national discrimination (11.3%), as well as ethno-national discrimination in combination with 1,2 and ≥ 3 other forms (17.2, 13.5 and 14.2%). Jewish immigrant mothers’ reports of these forms of discrimination were second highest, followed by non-immigrant Jewish mothers. However, non-immigrant Jewish mothers reported highest other forms of discrimination (not ethnic) compared to the other two groups (35.4%). This was followed by Jewish immigrant mothers (18.2%), and last by Palestinian-Arab mothers, of whom only 8% reported experiencing exclusive other forms of discrimination (not ethno-national).

As for the associations with PPD, we found that all forms of discrimination (SFD, MFD, and E-MFD) were significantly and positively associated with PPD (Table 2). Women who reported experiencing more forms of discrimination were more likely to have higher PPD. SFD were consistently associated with higher PPD. Highest prevalence of PPD was found among women who reported discrimination based on socioeconomic status (23.8%), and lowest PPD was found among mothers who reported discrimination based on gender (12.75%).

MFD was also associated with PPD in a dose-response manner. For the total sample, women who reported ≥3 forms of discrimination had the highest rates of PPD (18.4%), followed by women reporting 2 forms of discrimination (15.2%), then women reporting 1 form of discrimination (7.4%). Mothers reporting not experiencing any forms of discrimination had the lowest rates of PPD (6.0%) (Table 2).

As for the association between E-MFD and PPD, Table 2 shows that women who reported ethno-national discrimination combined with ≥3 other forms of discrimination had highest PPD prevalence (21.6%), followed by women reporting ethnic and 1 other form of discrimination (16.5% of PPD prevalence), then ethnic and 2 forms of discrimination (14.3%). Reporting exclusively ethno-national discrimination was associated with 10.2% PPD prevalence and reporting other forms of discrimination (not ethno-national) was associate with 9.5% prevalence of PPD among women.

Table 3 presents results of adjusted and unadjulsted multivariable logistic regression models (GEE) for the association between each of the discrimination measures (SFD, MFD and E-MFD) and PPD for each group of women. Regarding the multivariable association for each SFD measures and PPD, Table 3 shows that among non-immigrant Jewish women, only SFD based on age (AOR, 95%CI = 2.42, 1.25–4.71) and gender (AOR, 95%CI = 1.81, 1.09–3.02) were significantly associated with higher PPD in the adjusted model. Other SFD were not associated with PPD in non-immigrant Jewish women. Among Palestinian-Arab women, most of the SFD measures were associated with higher PPD except SFD based on ethno-national identity and religiosity level. In this group, a stronger association was observed for SFD based on socioeconomic status (AOR, 95%CI = 3.92, 1.74–8.84), and the weakest association for SFD based on gender (AOR, 95%CI = 1.86, 1.22–2.85). Among Jewish immigrant women, no significant association was found in the adjusted or unadjusted models between SFD and PPD except the association with discrimination based on ethnicity (AOR, 95%CI=3.46, 1.36-8.83).

**Table 3:** Logistic regressions for postpartum depression (PPD) and experiencing SFD, MFD, and E-MFD in the study groups of women: non-immigrant Jewish, Jewish immigrant and Palestinian-Arab (unadjusted and adjusted models)

	Non-immigrant Jewish (*N* = 621)		Immigrant Jewish (*N* = 214)		Palestinian-Arab (*N* = 260)	
	OR (95%CI)	AOR* (95%CI)	OR (95%CI)	AOR* (95%CI)	OR (95%CI)	AOR* (95%CI)
Single forms of discrimination (SFD)						
Discrimination based on ethnicity						
Yes	1.64 (0.83, 3.24)	1.75 (0.86, 3.54)	2.38 (0.91, 6.18)	3.46 (1.36, 8.83)	1.67 (0.99, 2.82)	1.70 (0.97, 2.99)
No	1	1	1	1	1	1
Discrimination based on socioeconomic status						
Yes	2.43 (1.13, 5.26)	2.45 (0.98, 6.08)	1.44 (0.51, 4.08)	1.71 (0.54, 5.40)	3.77 (1.76, 8.08)	3.92 (1.74, 8.84)
No	1	1	1	1	1	1
Discrimination based on religiosity level						
Yes	1.84 (0.90, 3.78)	1.85 (0.91, 3.74)	0.61 (0.15, 2.45)	0.73 (0.17, 3.04)	1.76 (1.02, 3.02)	1.66 (0.94, 2.95)
No	1	1	1	1	1	1
Discrimination based on age						
Yes	2.38 (1.20, 4.73)	2.42 (1.25, 4.71)	1.10 (0.23, 5.32)	1.04 (0.22, 4.95)	2.95 (1.14, 7.62)	2.87 (1.02, 8.07)
No	1	1	1	1	1	1
Discrimination based on gender						
Yes	1.59 (1.02, 2.49)	1.81 (1.09, 3.02)	0.80 (0.26, 2.43)	0.64 (0.19, 2.12)	1.98 (1.28, 3.05)	1.86 (1.22, 2.85)
No	1	1	1	1	1	1
Discrimination based on skin color						
Yes	2.49 (0.91, 6.81)	2.59 (0.98, 6.88)	0.88 (0.13, 5.78)	0.90 (0.16, 5.16)	2.80 (1.21, 6.44)	2.76 (1.14, 6.70)
No	1	1	1	1	1	1
Multiple forms of discrimination (MFD)						
≥ 3 forms	3.74 (1.53,9.16)	4.44 (1.74, 11.32)	1.99 (0.57, 6.91)	2.19 (0.57, 8.52)	2.60 (1.24, 5.49)	2.45 (1.15, 5.24)
2 forms	3.33 (1.20, 9.26)	3.67 (1.28, 10.47)	2.58 (0.74, 9.04)	2.91 (0.72, 11.72)	1.88 (0.79, 4.47)	1.91 (0.78, 4.69)
1 form	2.19 (0.87, 5.51)	2.62 (1.02, 6.76)	1.02 (0.23, 4.48)	1.26 (0.27, 6.01)	0.74 (0.25, 2.21)	0.78 (0.25, 2.40)
None	1	1	1	1	1	1
Ethnic and MFD combined (E-MFD)						
Other discrimination (not ethno-national)	2.94 (1.42, 6.11)	3.44 (1.64, 7.24)	0.07 (0.12, 5.36)	0.68 (0.10, 4.88)	1.76 (0.62, 5.01)	1.64 (0.62, 4.34)
Ethno-national and ≥ 3 other SFD	4.24 (1.66, 10.84)	5.02 (1.79, 14.03)	1.54 (0.20, 11.65)	2.06 (0.20, 21.74)	3.85 (1.69, 8.76)	3.95 (1.62, 9.64)
Ethno-national and 2 other SFD	2.49 (0.46, 13.50)	3.39 (0.67, 17.04)	1.83 (0.45, 7.46)	2.50 (0.60, 10.46)	1.41 (0.61, 3.24)	1.23 (0.51, 2.96)
Ethno-national and 1 other SFD	1.20 (0.18, 8.05)	1.29 (0.18, 9.15)	2.52 (0.52, 12.21)	2.65 (0.56, 12.55)	2.05 (0.91, 4.64)	2.06 (0.86, 4.95)
Ethno-national exclusive	3.11 (0.36, 26.97)	3.77 (0.39, 36.39)	3.13 (0.81, 12.02)	9.15 (1.61, 52.06)	0.42 (0.13, 1.38)	0.44 (0.11, 1.77)
None	1		1	1	1	1

*AOR- Adjusted for age, education, employment and antidepressant use

Table 3 also shows that MFD was strongly associated with PPD in non-immigrant Jewish women in a dose-response manner. Compared to non-immigrant Jewish women who reported no discrimination, those who reported ≥3 forms of discrimination were almost four and half times more likely to experience PPD (AOR, 95%CI = 4.4, 1.74–11.32); those who reported 2 forms of discrimination had almost four times more PPD (AOR, 95%CI = 3.67, 1.28–10.47); and reporting 1 form of discrimination was associated with almost three times more PPD (AOR, 95%CI = 2.62, 1.02–6.76). Among Palestinian-Arab women, the association between MFD and PPD was only significant among women who reported experiencing ≥3 forms of discrimination, which was associated with almost two and a half times more PPD compared to women who reported not experiencing any forms of discrimination (AOR, 95%CI = 2.45, 1.15–5.24). Among Jewish immigrant women no significant association was found between MFD and PPD in the multivariable analysis (Table 3).

Regarding the multivariable associations between E-MFD and PPD, Table 3 shows higher PPD among non-immigrant Jewish mothers who reported experiencing ethno-national discrimination combined with ≥3 other forms of discrimination (AOR, 95%CI =5.02, 1.79–14.03) and ‘any discrimination based on other grounds’ (not ethnic) (AOR, 95%CI = 3.44, 1.64–7.24), compared to Jewish women who reported not experiencing any form of discrimination. Among Jewish immigrant women, experiencing ethno-national discrimination only and not other forms was associated with almost 9 times higher PPD compared to Jewish immigrant mothers who reported no discrimination (AOR, 95%CI = 9.15, 1.61, 52.06). Among Palestinian-Arab women, the likelihood of PPD was almost 4 times higher (AOR, 95%CI = 3.95, 1.62–9.64) among women who reported experiencing ethno-national discrimination combined with ≥3 other forms of discrimination, compared to Arab women who reported experiencing no form of discrimination (Table 3).

The results we found when examining associations between SFD and MFD and E-MFD and PPD in each group of women informed our next step, in which we examined associations between composite MFD (C-MFD) and PPD. Table 4 presents multivariable results for PPD in association with C-MFD. C-MFD had stronger dose-response associations among Palestinian-Arab women, followed by Jewish immigrant women, then non-immigrant Jewish women. Compared to the reference group of non-immigrant Jewish women who reported no discrimination, PPD was higher among Palestinian-Arab women who reported ≥3, followed by Palestinian-Arab women reporting 2 forms of discrimination, then by Palestinian-Arab women reporting 1 form of discrimination (AOR, 95%CI = 12.68, 5.29–30.40, 10.08, 3.73–27.20, and 3.98, 1.23–12.86, respectively), as well as among Jewish immigrant women who reported ≥3 forms of discrimination (AOR, 95%CI = 4.44, 1.45–13.61), 2 forms of discrimination (AOR, 95%CI = 5.76, 1.84–17.97), and among non-immigrant Jewish women who reported experiencing ≥3, 2 and 1 forms of discrimination (AOR, 95%CI = 4.68, 1.87–11.71; 3.74, 1.32–10.63, and 2.70, 1.06–6.87, respectively). No significant association with PPD was found among Jewish immigrant women who reported one form of discrimination or no forms of discrimination. Notably, Palestinian-Arab who reported no discrimination were still more likely to experience PPD by almost fivefold (AOR, 95%CI = 4.76, 1.88–12.08) compared to non-immigrant Jewish women who reported no discrimination.

**Table 4:** Multivariable associations for postpartum depression (PPD) and composite multiple forms of discrimination (C-MFD*) in the total sample of mothers (*N* = 1095).

		Model 1		Model 2	
	*N*=	OR (95%CI)	*P*	OR (95%CI)	*P*
PA ≥ 3 forms	76	14.26 (6.27, 32.41)	< 0.001	12.68 (5.29, 30.40)	< 0.001
PA 2 forms	48	10.30 (4.06, 26.13)	< 0.001	10.08 (3.73, 27.20)	< 0.001
PA 1 form	43	4.06 (1.29, 12.77)	0.016	3.98 (1.23, 12.86)	0.021
PA none	93	5.47 (2.28, 13.12)	0.000	4.76 (1.88, 12.08)	0.001
JI ≥ 3 forms	50	4.21 (1.43, 12.41)	0.009	4.44 (1.45, 13.61)	0.009
JI 2 forms	39	5.45 (1.83, 16.26)	0.002	5.76 (1.84, 17.97)	0.003
JI 1 form	46	2.16 (0.56, 8.28)	0.263	2.32 (0.59, 9.12)	0.229
JI none	76	2.12 (0.69, 6.51)	0.191	2.22 (0.71, 6.98)	0.173
NIJ ≥ 3 forms	111	3.74 (1.53, 9.16)	0.004	4.68 (1.87, 11.71)	0.001
NIJ 2 forms	73	3.33 (1.20, 9.26)	0.021	3.74 (1.32, 10.63)	0.013
NIJ 1 form	151	2.19 (0.87, 5.51)	0.096	2.70 (1.06, 6.87)	0.038
NIJ 0 none	289	1.00		1.00	

* Interactions between women’s identity and MFD,

*PA* Palestinian-Arab women,

*JI* Jewish immigrant women,

*NIJ* Non-immigrant Jewish women,

Model 1- unadjusted, Model 2- adjusted for age, education, employment and antidepressant use

## Discussion

Our study drew on intersectionality theory [2, 11, 18] to compare relationships between different measures of discrimination (SFD, MFD, E-MFD and C-MFD) and PPD among Indigenous minority, immigrant, and majority groups of mothers in Israel (Palestinian-Arab, Jewish immigrant, and non-immigrant Jewish). To calculate these measures of discrimination, we first asked the study participants about six SFD (discrimination based on ethno-national identity, socioeconomic status, religiosity level, age, gender, and skin color), then calculated the sum score of reports of experiencing these forms of discrimination (0,1,2 and ≥ 3 forms). Since ethno-national discrimination is at the core of experiencing discrimination, we calculated a variable that included experiencing ethno-national discrimination combined with other forms of discrimination (E-MFD). Lastly, we created a composite MFD variable (C-MFD) that included interactions between MFD and women’s identity (Palestinian-Arab minority, Jewish immigrant, and non-immigrant Jewish citizens of Israel).

We found that Palestinian-Arab minority women reported higher SFD, MFD and E-MFD. This was followed by immigrant Jewish women, while non-immigrant Jewish women reported these least often. These results coincide with previous research on multiple discrimination among minority women [18, 21, 40, 63], and with research in Israel showing that Palestinian-Arab citizens experience discrimination in various facets of life [22, 44, 64] and are more stigmatized compared to the two Jewish groups (immigrant and non-immigrant women) [46]. The unexpected non-significant differences between the study groups regarding gender-based discrimination might relate to different perceptions and levels of awareness about this form of discrimination among women in these diverse cultural groups. While Palestinian-Arab women face higher gender imbalances and even higher intimate partner violence, they reported lower gender discrimination than the other two groups of Jewish women. We think this might reflect tolerance for, or ignorance about gender based discrimination in this group, as sexist behavior and beliefs pervade their community. The women may have internalized this ideology and may perceive it as part of the consensus that builds Palestinian Arab community collective efficacy [38].

Prevalence of PPD was also higher among Palestinian-Arab minority women (20.8%) compared to Jewish immigrant women (9.1%) and non-immigrant Jewish women (6.2%). This result was also obtained in our previous research on PPD in Israel using the current study data [36], and elsewhere [35].

A main finding of the current study was that while most SFD showed a strong association with PPD for Palestinian-Arab women, MFD and E-MFD only had a strong dose-response relationship with PPD among non-immigrant Jewish mothers. Only Palestinian-Arab mothers who reported experiencing high MFD (≥3 forms of discrimination) and high E-MFD (ethno-national discrimination and ≥ 3 other forms of discrimination) were more likely to have higher PPD. Among Jewish immigrant mothers, no association was found between SFD, MFD, E-MFD and PPD except for discrimination based on ethnic identity. These results partially confirm our hypothesis regarding a strong relationship between MFD and PPD in minority mothers (Palestinian-Arab and Jewish immigrant study groups). Emerging research in several contexts has shown associations among minorities between MFD and poor mental health outcomes [8, 23, 63, 65]. Our results among Palestinian-Arab mothers show that it’s only when these women experience more severe discrimination (≥3 forms of discrimination, and ethno-national discrimination combined with ≥3 other forms of discrimination) that there is an association with higher PPD, and therefore a likelihood that women’s health has suffered. This might indicate that Palestinian-Arab women have developed resilience or tolerance to lower levels of discrimination, given the longstanding history of discrimination against the Palestinian-Arab ethno-national minority in Israel. We also know that both institutional and interpersonal discrimination were detrimental to mental health of Arab men [66].

Among Jewish immigrant women, the picture was different. We found that only the measure of experiencing exclusively ethnic discrimination (and not other forms or combinations) was associated with more PPD—9 times more. The other discrimination measures had no association with PPD for this group. This might indicate a tremendous impact on Jewish immigrant women’s mental health and PPD when they feel themselves to be outside the consensus on Israeli Jewish ethnic identity and are discriminated against on the basis of ethnicity. Previous research on discrimination and health in Israel showed that while Arabs and immigrants reported higher experiences of discrimination, there was no significant association between discrimination and health in these groups [56]. Our results suggest that in order to paint a fuller picture of the impact of discrimination on health and mental health, future research in this area should consider MFD and the intensity, for each of these population groups, of experiencing these forms.

A second main finding of this study was the dose-response relationship that emerged through the use of the composite measure of interactions between MFD and women’s identity (C-MFD). The C-MFD, which had 12 categories, revealed that associations with PPD changed in a dose-response manner within and between study groups compared to the reference category of non-immigrant Jewish women who reported no discrimination. Palestinian-Arab women who reported experiencing ≥3 forms of discrimination had highest PPD (AOR was almost 12), followed by Palestinian-Arab women who reported experiencing 2 forms of discrimination (AOR = 10), then Palestinian-Arab women who reported one form of discrimination (AOR almost 4). Among Jewish immigrant women, those who reported experiencing 2 forms of discrimination had highest PPD (AOR almost 6), followed by Jewish immigrant with experiencing ≥3 forms of discrimination (AOR of 4.4), compared to non-immigrant women who reported no discrimination. Lastly, among non-immigrant Jewish women, those to who reported experiencing ≥3 forms of discrimination (AOR = 4.7) had highest PPD, followed by those who reported 2 forms of discrimination (AOR = 3.7), then those who reported one form of discrimination (AOR = 2.7), compared to non-immigrant Jewish women who reported no discrimination.

The results of our use of the composite C-MFD measure also confirmed our hypothesis regarding its impact among Palestinian-Arab minority and Jewish immigrant women. This lends support to intersectionality theory, which advances the idea that multiple marginalized axes of identity (the grounds for the MFD reported by women in the study) intersect with ethno-national identity to produce stronger effects on health than can be accounted for by looking only at cumulative effects of SFD. In this study, ethno-national identities interacted with MFD to reveal social inequalities in PPD. The C-MFD measure allowed us to compare privilege or lack thereof among the three groups of women based on facets of their identity and their reports of the grounds they perceive for discrimination (0,1,2, ≥3 forms of discrimination). Our work suggests that using an additive measures of MFD might underestimate the effects of multiple discrimination on maternal mental health among minority women—here, Palestinian-Arab minority women and Jewish immigrant women—since, among these women, the additive MFD measure without interaction with women’s identity showed a weak association with PPD in Palestinian-Arab women, and no associations with PPD in Jewish immigrant women.

Indeed, results on the association between C-MFD and PPD also align with previous research showing that minority groups [15] and minority women suffer multiple forms of oppression [10]. This supports Bowleg’s suggestion that interactions should be used to capture the complexity of interlocking exposures, such as discrimination, rather than using an additive measure or sum of different forms of marginalization [18]. We used composite-MFD, which included an interaction between our main exposure (MFD) and ethno-national identities of women. Our analytic strategy revealed social inequalities in PPD between the 12 groups of women. The greatest inequality was experienced by the marginalized group of Palestinian-Arab women, followed by Jewish immigrant women, and last by non-immigrant Jewish women. This echoes previous findings on stigmatization among segments of Israeli society [46]. It is clear that Palestinian-Arab minority women suffer multiple marginalization [67]: as members of the Palestinian-Arab minority, as women in a patriarchal society, and as residents in low socioeconomic status neighborhoods [54, 55]. This might have led our participants to report experiencing more MFD. This would be unsurprising, as previous research has reported higher ethnic discrimination towards Palestinian-Arabs compared to Jewish Israelis [56]. As well, for many years, Palestinian-Arabs have been geographically segregated, with 85% residing in low-income towns and villages [54, 55], and, as mentioned earlier, Palestinian-Arabs in Israel also suffer from discriminatory institutional policies, and this has been associated with low socioeconomic status and poor mental health [22].

As for Jewish immigrant women in Israel, experiences of MFD might be complex, and should be examined in future research by country of origin, as this may shed light on differences in experiences of discrimination and level of marginalization among them in Israeli society [42, 46]. No differences in PPD among Jewish immigrant women in relation with MFD might have resulted from contrasting reports on MFD among Jewish immigrant groups depending on their country of origin, given that, in Israel, country of origin among immigrant Jews determines resources and opportunities [42].

### Study strength and limitations

This is the first study we know of to examine associations between multiple forms of discrimination (MFD) and PPD among three groups of mothers: Indigenous ethnic minority, immigrant and non-immigrant majority. However, there are some study limitations. First, the study sample is made up of mothers visiting MCH clinics in Israel. This could have introduced a selection bias. Yet, few mothers of reproductive age in Israel fail to visit MCH clinics, as almost all children receive follow-ups and immunizations in these clinics. Further, the use of a stratified sample while taking into account proportions of births by district and ethno-national group composition (Palestinian-Arab and Jewish) was a study strength, as the sample’s socio-demographic characteristics were similar to those of women of reproductive age in Israel [58]. Second, since the current study was cross-sectional and the measures of discrimination were subjective, it is possible that mothers who have PPD reported more discrimination. However, longitudinal studies have shown that discrimination pre-dates mental health issues among mothers (and fathers) [26]. Another limitation of the work is that our measure of discrimination is subjective. It is possible that the mothers who are oppressed and not oppressed have different perceptions and interpretation of similar events. This paradox should be considered when synthesizing the results for women from different cultures. At the same time, many measures of discrimination that we know are subjective, based on individual perceptions of events. Our measures asked about the grounds of discrimination (based on ethnicity, age, skin color, religiosity level, or gender). and not whether it happened to them or not. In addition, the discrimination measures in the current study did not consider the time and context of discrimination experiences. Future research should ask about the type of discrimination as well as the time and length of experiences of discrimination as a means of better understanding its possible cumulative effects on mental health [15, 26]. Finally, the measures of SFD used in this study asked about 6 grounds of discrimination, but did not include others, such as disability, body weight, and sexual orientation, which have been used in previous research for measuring MFD [15]. Future research should include these facets of identity as important forms of discrimination. Finally, we excluded pregnant women from the current analysis. Future research should look into associations between MFD during pregnancy and PPD after birth.

## Conclusions

Results show that a composite measure of MFD (C-MFD) and women’s ethno-national identity had a stronger dose-response relationship with PPD depending on experiencing different grounds of discrimination. Use of this measure revealed large inequalities between the study ethno-national groups. This result highlights the importance in maternal health research of studying social identities in intersection with MFD. Health care professionals should consider MFD in prevention and treatment of PPD, especially among mothers located at more than one marginalized axis of identity.

### Supplementary information

**Additional file 1.** Correlations between the study variables

## Data Availability

The datasets generated and analyzed during the current study are not publicly available due to legal restrictions. For data requests, please contact the PI: Dr. Nihaya Daoud at: daoud@bgu.ac.il

## Appendix 1

**Table 5:** Correlations between the study variables

	1	2	3	4	5	6
1. Postpartum depression (PPD)	1.00	0.18^***^	−0.11^***^	−0.08^***^	0.14^***^	0.09^***^
2. Women’s study groups		1.00	−0.02	−0.27^***^	0.24^***^	0.37^***^
3. Antidepressant use			1.00	−0.06^**^	0.01	−0.02
4. Age				1.00	−0.20^***^	−0.18^***^
5. Women’s education					1.00	0.33^***^
6. Employment status						1.00

Significant level: ***: *p* ≤ 0.001; **: 0.001 < *p* ≤ 0.01

## Abbreviations

C-MFD: Composite MFD that interacted with women’s identity.
E-MFD: Ethnic discrimination combined with other MFD (not ethnic)
MFD: Multiple forms of discrimination
PPD: Postpartum depression
SFD: Single forms of discrimination
