# Supplementary material for: Multiple forms of discrimination and postpartum depression among indigenous Palestinian-Arab, Jewish immigrants and non-immigrant Jewish mothers
Source: BMC Public Health. 2019 Dec 27;19:1741. doi: 10.1186/s12889-019-8053-x (PMC6935055; doi:10.1186/s12889-019-8053-x)
Supplement: Supplementary file 1 — Additional file 1. Correlations between the study variables [file 12889_2019_8053_MOESM1_ESM.docx]

**Appendix 1- Correlations between the study variables**

|  | **1** | **2** | **3** | **4** | **5** | **6** |
| --- | --- | --- | --- | --- | --- | --- |
| **1. Postpartum depression (PPD)** | 1.00 | 0.18^***^ | -0.11^***^ | -0.08^***^ | 0.14^***^ | 0.09^***^ |
| **2. Women’s study groups** |  | 1.00 | -0.02 | -0.27^***^ | 0.24^***^ | 0.37^***^ |
| **3. Antidepressant use** |  |  | 1.00 | -0.06^**^ | 0.01 | -0.02 |
| **4. Age** |  |  |  | 1.00 | -0.20^***^ | -0.18^***^ |
| **5. Women's education** |  |  |  |  | 1.00 | 0.33^***^ |
| **6. Women currently working** |  |  |  |  |  | 1.00 |

Significant level: ***: p ≤0.001; **: 0.001 < p ≤ 0.01
